# Predicting survival time of lung cancer patients using radiomic analysis

**DOI:** 10.18632/oncotarget.22251

**Published:** 2017-11-01

**Authors:** Ahmad Chaddad, Christian Desrosiers, Matthew Toews, Bassam Abdulkarim

**Affiliations:** ^1^ Division of Radiation Oncology, McGill University, Montréal, Canada; ^2^ The Laboratory for Imagery, Vision and Artificial Intelligence, Ecole de Technologie Supérieure, Montréal, Canada

**Keywords:** lung cancer, NSCLC, cancer staging, radiomics, texture features

## Abstract

**Objectives:**

This study investigates the prediction of Non-small cell lung cancer (NSCLC) patient survival outcomes based on radiomic texture and shape features automatically extracted from tumor image data.

**Materials and Methods:**

Retrospective analysis involves CT scans of 315 NSCLC patients from The Cancer Imaging Archive (TCIA). A total of 24 image features are computed from labeled tumor volumes of patients within groups defined using NSCLC subtype and TNM staging information. Spearman’s rank correlation, Kaplan-Meier estimation and log-rank tests were used to identify features related to long/short NSCLC patient survival groups. Automatic random forest classification was used to predict patient survival group from multivariate feature data. Significance is assessed at *P* < 0.05 following Holm-Bonferroni correction for multiple comparisons.

**Results:**

Significant correlations between radiomic features and survival were observed for four clinical groups: (group, [absolute correlation range]): (large cell carcinoma (LCC) [0.35, 0.43]), (tumor size T2, [0.31, 0.39]), (non lymph node metastasis N0, [0.3, 0.33]), (TNM stage I, [0.39, 0.48]). Significant log-rank relationships between features and survival time were observed for three clinical groups: (group, hazard ratio): (LCC, 3.0), (LCC, 3.9), (T2, 2.5) and (stage I, 2.9). Automatic survival prediction performance (i.e. below/above median) is superior for combined radiomic features with age-TNM in comparison to standard TNM clinical staging information (clinical group, mean area-under-the-ROC-curve (AUC)): (LCC, 75.73%), (N0, 70.33%), (T2, 70.28%) and (TNM-I, 76.17%).

**Conclusion:**

Quantitative lung CT imaging features can be used as indicators of survival, in particular for patients with large-cell-carcinoma (LCC), primary-tumor-sizes (T2) and no lymph-node-metastasis (N0).

## INTRODUCTION

Lung cancer is the most frequently diagnosed type of cancer and the leading cause of cancer-related deaths worldwide [1]. It can be divided in two main categories: non-small cell lung cancer (NSCLC) and small cell lung cancer (SCLC). NSCLC is the most prevalent type of lung cancer, accounting for approximately 85% of cases [2], and can usually be labeled as squamous cell carcinoma, large cell carcinoma, adenocarcinoma or not otherwise specified (NOS). Squamous cell carcinoma, which accounts for 25% of all lung cancers, generally occurs in the center of the lung and is often associated with smokers. On the other hand, large cell carcinoma (LCC) is a rapid growing tumor that can occur anywhere in the lung and represents about 10% of NSCLC cases. Adenocarcinoma, which accounts for half of NSCLC cases, is a slower-growing type of lung cancer often seen peripherally in the lungs. Although more frequent in smokers, adenocarcinoma is also the most common form of lung cancer in non-smokers [3, 4]. Finally, NOS corresponds to less frequent NLCSC subtypes or cases for which a more specific diagnosis cannot be made.

The progression of lung cancer is typically described using five stages (0 to IV), ranging from a tumor limited to the lining layer of airways (Stage 0) to a cancer that has spread to lymph nodes and major organs in the body (Stage IV). The accurate staging of lung cancer is essential to establish prognosis and select an optimal treatment plan (e.g., surgery, chemotherapy and/or radiotherapy). However, staging information is not necessarily predictive of disease progression or response to treatment.

In recent years, image analysis techniques have been used successfully to provide personalized prognosis and treatment plans with a greater accuracy. In particular, radiomics analysis methods, which describe a segmented tumor region using various quantitative features derived from image data, have shown a great potential for predicting survival outcome of patients with lung cancer [5-10], colorectal cancer [11, 12], or brain tumors [13-15].

Several studies have investigated the relationship between image features and lung cancer. Ganeshan et al. showed that texture features extracted from CT images of lung tumors were correlated with glucose metabolism and lung cancer stages [16]. Various texture features, including those based on intensity histograms, absolute gradients, nearest grey tone difference matrices (NGTDM), grey-level co-occurrence matrices (GLCM), Laplacian of Gaussian (LoG) filtration and wavelets, have also been proposed to predict the survival group (e.g., below or above median survival) of patients with NSCLC [17-20]. In Ganeshan et al [21], LoG features derived from CT scans were shown to predict the survival time of NSCLC patients more accurately than fluorodeoxyglucose (FDG) uptake in positron emission tomography (PET). Likewise, shape features have also been used to assess NSCLC prognosis. In Tixier *et al* [22], high tumor volume was found to be associated with short survival time in a population of NSCLC patients treated with surgery and chemotherapy. Tumor compactness, asymmetry and location have also been linked with the survival outcome of NSCLC patients [17]. In Aerts *et al* [7], a large number extracted features from CT data were shown to have prognostic power in independent data sets of lung and head-and-neck cancer. Shape and texture features extracted from CT images were also used for the detection of lung nodules and their characterization as benign or malignant [23-27].

Currently, the application of imaging features for the prediction of survival in NSCLC subtypes (i.e., large cell carcinoma, squamous cell carcinoma and adeno-carcinoma) and in individual TNM stages is still relatively limited. Since the histological properties and proliferation mechanisms of these subtypes and stages are quite different, analyzing them individually could provide a more accurate and personalized prognosis, thereby leading to better therapeutic plans.

This study aims to investigate the usefulness of diverse texture and shape features for predicting the survival outcome of patients with specific NSCLC subtypes and TNM stages. To our knowledge, this work is the first radiomics-based study to analyze survival within these specific patient groups, showing that the relevance of these features varies significantly from a patient group to another.

## RESULTS

We start by describing the demographics of patients used in our study. Afterwards, we summarize the results of the univariate and multivariate analyses proposed to evaluate the informativeness of radiomic features in predicting NSCLC survival outcome. A detailed description of the data and the proposed radiomic analyses can be found in the Materials and methods section.

### Patient characteristics

All 315 NSCLC patients were grouped based on histology and TNM classification (Table 1). Among them, 277 (n = 96 censored) patients were grouped in four histology classes with median (IQR) age of LCC = 65.20 (59.26–74.06), SCC = 70.79 (64.01–78.71), ADC = 64.58 (59.79–74.45) and NOS = 65.79 (56.88–74.86); 312 (n = 107 censored) patients were grouped in four tumor size with median (IQR) age of T1 = 68.43 (61.44–75.01), T2 = 69.78 (60.03–77.11), T3 = 63.88 (58.35–76.79) and T4 = 64.61 (57.51–72.63); 315 (n = 108 censored) patients were grouped in five lymph nodes type with median (IQR) age of N0 = 70.47 (60.71–78.71), N1 = 71.78 (61.77–74.08), N2 = 65.02 (58.39–72.82), N3 = 62.86 (54.70–70.64) and N4 = 73.28 (66.21–74.92); 315 (108 censored) patients were grouped in four distant metastases, primarily M0 with median (IQR) age of M0 = 67.27 (59.42–75.26); 314 (n = 108) patients were grouped in four TNM group with median (IQR) age of I = 71.94 (64.56–79.27), II = 74.73 (61.09–78.77), IIIa = 66.91 (59.38–73.07), IIIb = 64.18 (56.59–71.05).

**Table 1:** Demographic information for NSCLC patients

Groups	*n* (censored)	Male	Female	Age	
				(avg ± stdev)	Median (IQR)
**NSCLC subtype**					
LCC	100 (40)	62	38	66.86 ± 15.14	65.20 (59.26–74.06)
SCC	90 (28)	74	16	70.98 ± 15.99	70.79 (64.01–78.71)
ADC	31 (9)	20	11	66.37 ± 15.09	64.58 (59.79–74.45)
NOS	56 (19)	42	14	65.83 ± 19.85	65.79 (56.88–74.86)
**T stage**					
T1	73 (32)	46	27	61.02 ± 24.52	68.43 (61.44–75.01)
T2	120 (36)	91	29	65.92 ± 18.16	69.78 (60.03–77.11)
T3	38 (13)	29	9	64.78 ± 15.58	63.88 (58.35–76.79)
T4	81 (26)	58	23	62.67 ± 15.57	64.61 (57.51–72.63)
^*^	2 (1)	–	–	–	–
**N stage**					
N0	133 (50)	101	32	65.78 ± 21.04	70.47 (60.71–78.71)
N1	14 (3)	11	3	61.57 ± 26.80	71.78 (61.77–74.08)
N2	97 (28)	64	33	62.61 ± 17.49	65.02 (58.39–72.82)
N3	68 (25)	46	22	60.55 ± 16.17	62.86 (54.70–70.64)
N4	3 (2)	3	0	71.47 ± 4.63	73.28 (66.21–74.92)
**M stage**					
M0	311 (108)	221	90	63.72 ± 19.01	67.27 (59.42–75.26)
M1	1 (0)	1	0	–	–
M2	0	0	0	–	–
M3	3 (0)	3	0	65.87 ± 11.1	71.47 (53.08–73.05)
**Grouping TNM**					
I	81 (30)	60	21	64.96 ± 24.64	71.94 (64.56–79.27)
II	26 (7)	21	5	68.05 ± 21.88	74.73 (61.09–78.77)
IIIa	73 (24)	48	25	64.57 ± 14.60	66.91 (59.38–73.07)
IIIb	134 (47)	95	39	61.27 ± 17.11	64.18 (56.59–71.05)
^*^	1	–	–	–	–

^*^Unknown; *n* is the number of subjects; censored is the number of subjects that their survival from time of scans to last visit.

### Correlation analysis

**Figure 1:**
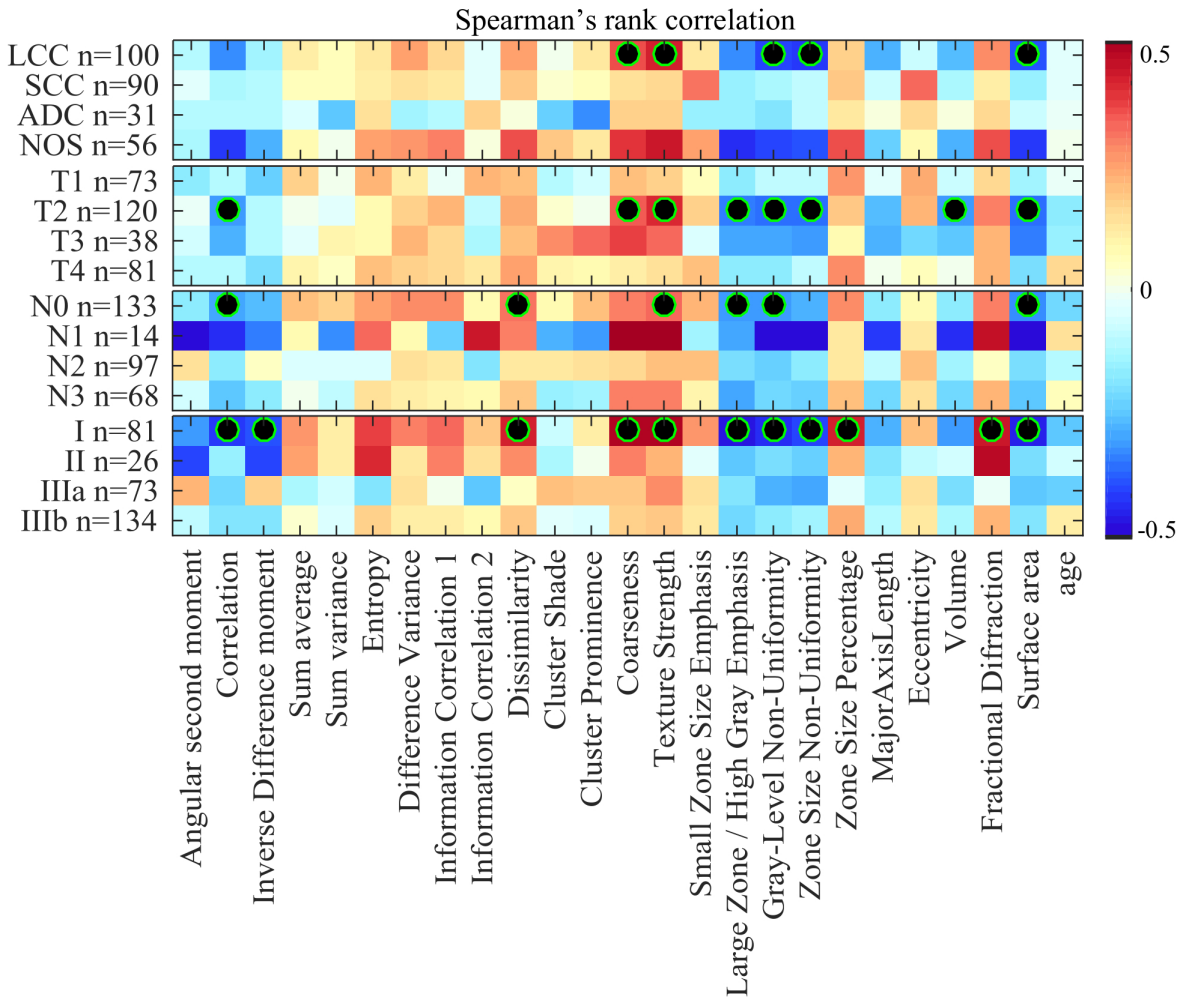
Heatmap of Spearman rank correlation between feature value and survival time, color-coded from -0.5 (dark blue) to 0.5 (dark red) Censored patients were considered using an imputation strategy which computes their survival time as the mean survival of deceased patients with time-to-death greater or equal to censored patients’ time of last visit. Features with statistically significant correlation (i.e., corrected *P* < 0.05) are indicated with a black-green circle.

Figure 1 shows the Spearman rank correlation between radiomic features (plus age) and the survival time of patients, for groups defined using NSCLC subtype and TNM variables. With respect to patient groups defined based on NSCLC subtypes, we observe the highest absolute correlation values for patients with large cell carcinoma (LCC) and not other specified (NOS) subtypes. In particular, five features appear to be moderately correlated with the survival time of LCC patients (i.e., coarseness, texture strength, grey-level non-uniformity, zone size non-uniformity and surface area), with absolute correlation values between 0.35 and 0.43. These results are statistically significant with corrected *P* < 0.05. Correlation values for NOS patients are not statistically significant following Holm-Bonferroni correction, although this could be due to the smaller number of patients in that group. Note that the SCC group shows no significance, while having a size similar to LCC.

In the case of groups related to tumor size (T), we also find eight radiomic features moderately correlated with survival for patients with T2 tumors (i.e., correlation, coarseness, texture strength, large zone/high grey emphasis, grey-level non-uniformity, zone size non-uniformity, volume and surface area), with absolute correlation values between 0.31 and 0.37. Features derived from T1, T3 and T4 tumors exhibit lower correlation values that are not significant following Holm-Bonferroni correction.

For patient groups derived from lymph node (N) variables, six features (i.e., correlation, dissimilarity, texture strength, large zone/high grey emphasis, grey-level non-uniformity and surface area) are moderately correlated to survival time for patients without lymph node involvement (N0), with absolute correlation values around 0.30. Features derived from N1 patients also show mild correlations. However, these are not significant following Holm-Bonferroni correction, possibly due to the small size of this group. In contrast, N2 and N3 groups have comparably weaker correlation values than N0, none of these values being statistically significant. Unlike for N1, group size is not such an important factor in these results of non-significance (97 and 68 patients with N2 and N3, respectively).

Finally, our analysis within groups based on overall stage reveals 11 radiomic features moderately correlated with survival in Stage I patients (i.e., correlation, inverse different moment, dissimilarity, coarseness, texture strength, large zone/high grey emphasis, grey-level non-uniformity, zone size non-uniformity, zone size percentage, fractional diffraction and surface area) with absolute correlation values between 0.39 and 0.49. Lower correlation values were found in Stage II, IIIA and IIIB groups, which are not significant following Holm-Bonferroni correction.

Overall, our correlation analysis finds the strongest associations between radiomic features and patient survival for LCC (and potentially NOS), T2, N0 and Stage I groups. These groups correspond mainly to large cell carcinoma cancers with primary tumor size between 3 cm and 7 cm across [28, 29] and no evidence of regional lymph node involvement or distant metastasis. Our analysis also reveals a subset of radiomic features exhibiting significant correlation values across various groups. In particular, features corresponding to texture strength, grey-level non-uniformity and surface area were found significant in all four of the LCC, T2, N0 and Stage I groups.

To rule out censorship as possible confound in our analysis, we also computed the Spearman rank correlation between radiomic features and survival time of uncensored patients. Results, which can be found in Figure 2 of Supplementary Materials, are consistent with those obtained via our imputation strategy: moderate correlation is observed for almost the same radiomic features in LCC, T2, N0 and Stage I groups.

**Figure 2:**
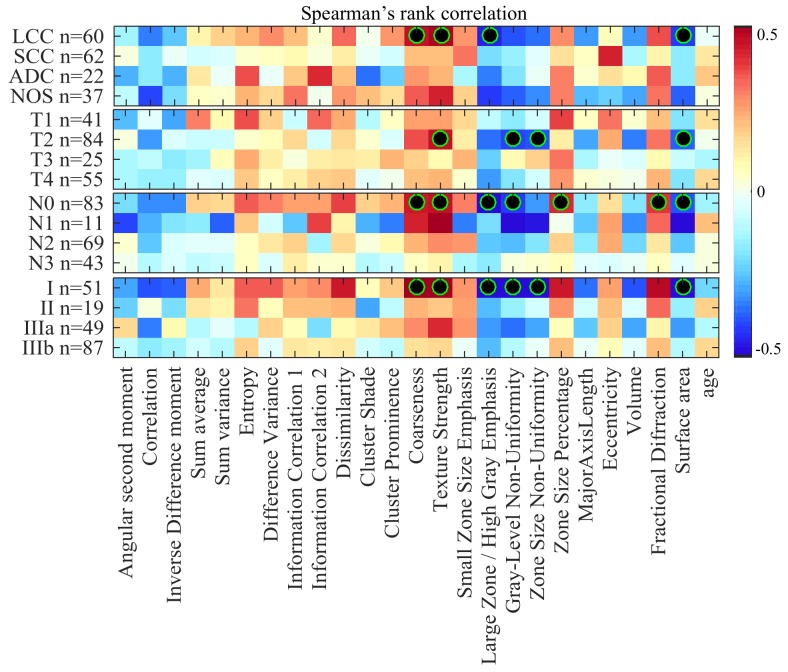
Heatmap of Spearman rank correlation between feature value and survival time of uncensored (i.e., deceased) patients, color-coded from -0.5 (dark blue) to 0.5 (dark red) Features with statistically significant correlation (i.e., corrected *P* < 0.05) are indicated with a black-green circle.

### Kaplan-Meier survival analysis

**Figure 3:**
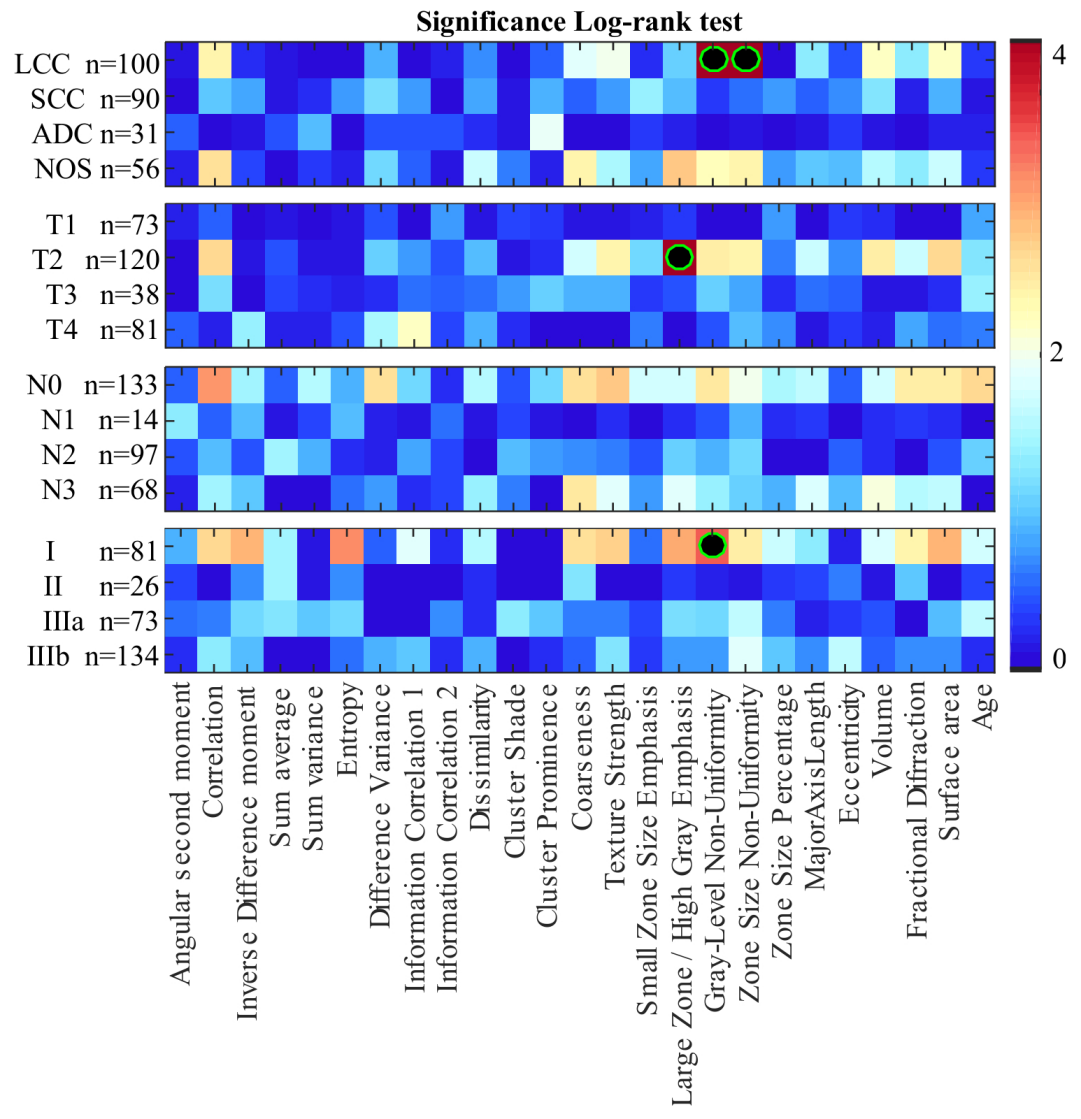
Heatmap of log-rank test p-values (–log_10_ scale) using features to separate patients in two groups: those with feature values less than the median, and those with values above or equal to the median Features leading to groups with significantly different survival profiles (i.e., corrected *P* < 0.05) are indicated with a black-green circle.

**Figure 4:**
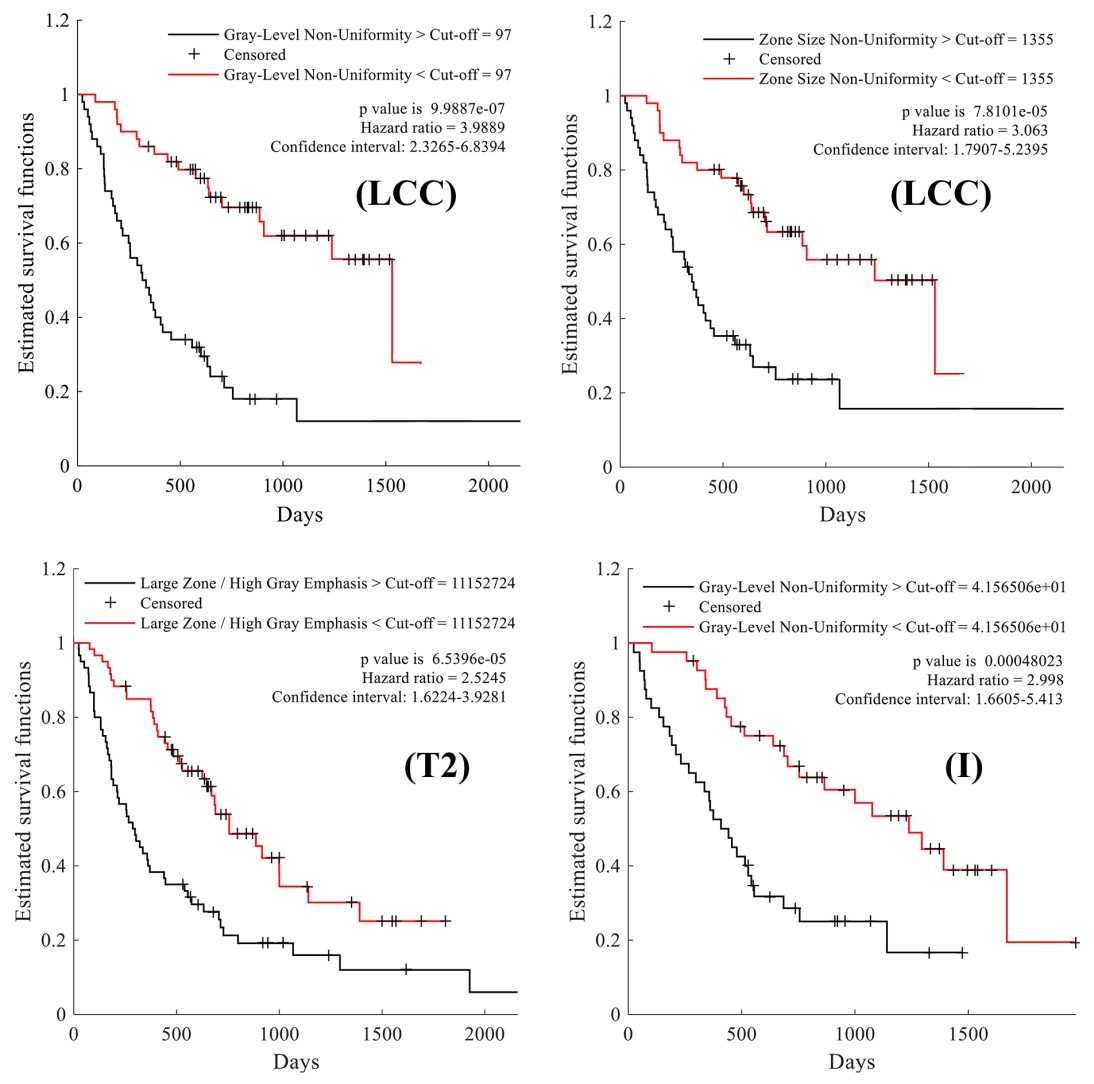
Kaplan-Meier survival curves comparing the survival rate of patient groups obtained using median feature values as cut-off Large cell carcinoma (LCC) patients split using the median value of the following texture features: grey-level non-uniformity and zone size non-uniformity, respectively. Patients with tumor size of T2 and Stage I cancer, separated based on the median value of the following texture features: large zone/high grey emphasis and grey-level non-uniformity, respectively.

The results of our survival analysis based on the Kaplan-Meier estimator and log-rank test are summarized in Figure 3, Figure 4 and Table 2.

**Table 2:** Kaplan-Meier survival analysis of the NSCLC clinical factors known to correlate with survival.

				Median survival (Month)								
Feature	Cut-off (median)			Above cut-off			Below cut-off			p*-*value^*^		
	LCC	T2	Stage I	LCC	T2	Stage I	LCC	T2	Stage I	LCC	T2	Stage I
*f*_1_ ×10^-3^	310	332	222	18.87	17.50	15.90	18.77	15.47	25.30	1	1	1
*f*_2_ ×10^-3^	822	841	796	11.53	10.97	16.57	24.33	20.13	24.97	0.43	0.237	0.251
*f*_3_ ×10^-3^	732	722	608	16.93	17.73	14.60	19.40	15.47	30.10	1	1	0.144
*f*_4_ ×10^-1^	310	331	427	19.03	14.13	25.07	17.43	18.23	15.97	1	1	1
*f*_5_×10^0^	1559	1642	1955	18.87	14.77	17.83	18.77	17.73	18.50	1	1	1
*f*_6_ ×10^-3^	290	308	398	18.40	15.07	31.60	19.03	18.23	14.43	1	1	0.078
*f*_7_×10^0^	127	119	168	21.23	16.83	21.43	14.97	14.73	18.10	1	1	1
*f*_8_ ×10^-3^	-299	-317	-277	21.13	16.00	23.17	15.73	15.63	15.97	1	1	1
*f*_9_ ×10^-3^	785	797	818	18.77	15.37	22.37	18.90	18.20	17.63	1	1	1
*f*_10_ ×10^-3^	4565	4085	6871	21.23	16.00	25.23	14.97	15.57	17.17	1	1	1
*f*_11_×10^0^	38774	43180	29783	18.40	16.00	17.57	19.17	16.43	21.30	1	1	1
*f*_12_ ×10^4^	540	544	610	21.77	16.83	17.57	14.27	15.20	18.50	1	1	1
*f*_13_ ×10^-5^	250	200	000	21.63	17.37	28.50	11.10	11.77	15.23	1	1	0.282
*f*_14_×10^-3^	909	849	2543	21.63	18.53	26.73	12.93	10.03	15.23	0.93	0.432	0.203
*f*_15_×10^-3^	734	725	771	20.30	18.07	24.20	17.43	12.50	15.97	1	1	1
*f*_16_×10^4^	600	111	116	11.53	9.77	15.00	21.77	20.90	25.30	1	**0.007**	0.131
*f*_17_ ×10^0^	97	117	41	10.83	10.97	14.20	23.50	18.53	27.27	**< 0.001**	0.381	**0.048**
*f*_18_×10^0^	1355	1743	697	11.40	10.97	15.00	23.50	20.13	27.27	**0.007**	0.432	0.363
*f*_19_×10^-3^	80	83	157	20.23	16.77	25.23	17.43	12.43	15.83	1	1	1
*f*_20_×10^-2^	5382	6161	3585	13.17	12.13	15.60	21.23	18.33	25.30	1	1	1
*f*_21_×10^-3^	997	997	996	17.27	18.33	18.33	19.40	14.07	17.63	1	1	1
*f*_22_×10^0^	10617	14314	3837	12.13	10.97	15.60	23.23	19.17	25.30	0.6	0.364	1
*f*_23_×10^-3^	2629	2607	2766	21.23	18.63	28.50	12.13	10.97	15.23	1	1	0.419
*f*_24_×10^0^	5949	6205	1984	12.50	10.97	15.00	22.50	19.17	28.13	0.64	0.264	0.131
Age	65.2	69.78	71.94	15.53	14.67	14.60	19.30	19.03	24.10	1	1	1
^*^ Following Holm-Bonferroni correction.												

Figure 3 gives the log-rank test significance (in –log_10_ *P*, where *P* is the corrected p-value) obtained by splitting patients with the median value of each feature (i.e., the cut-off). Values higher than 1.30 correspond to features whose median separates patients in groups having significantly different survival profiles, with *P* < 0.05 following Holm-Bonferroni correction. As in the previous analysis, significance is measured within patient groups associated to NSCLC subtypes and TNM staging variables. For NSCLC subtypes, two features show significantly different survival distributions when dividing LCC patients based on their median value: grey-level non-uniformity and zone size non-uniformity. Likewise, the large zone/high grey emphasis feature derived from patients with tumor size T2 and the grey-level non-uniformity feature in Stage I patients result in significantly different survival profiles. We note that these four features were also found to be statistically correlated with survival in the previous analysis.

Table 2 reports, for each feature derived from LCC, T2 and Stage I patient groups, the median feature value used as cut-off, the median survival time of patients with values below and greater to this cut-off, and the log-rank p*-*value following Holm-Bonferroni correction. The four features yielding significant differences in their respective patient group are highlighted using bold underlined font. Figure 4 presents the Kaplan-Meier curves obtained using the cut-off value of these features. We observe that LCC patients with below-median values of grey-level non-uniformity have a higher survival rate, with a hazard ratio (HR) of 3.9 and a median survival of 705 days compared to 325 days for other LCC patients. Likewise, LCC patients with below-median values of zone-size non-uniformity have better survival odds, with a hazard ratio of 3.06 and median survival time of 705 days compared to 342 days for other patients in this group. Similarly, we see that patients in the T2 group with below-median values of large zone/high grey emphasis have a higher survival rate with a hazard ratio of 2.5 and a median survival of 627 days compared to 293 days for other patients in this group. Finally, we find that Stage I patients with below-median values of grey-level non-uniformity have higher survival time, with a hazard ratio of 2.99 and median survival time of 818 days compared to 426 days for other Stage I patients. In summary, this analysis confirms previous results that texture features derived from LCC, T2, and Stage I patient groups are associated with NSCLC survival.

### Survival prediction

**Figure 5:**
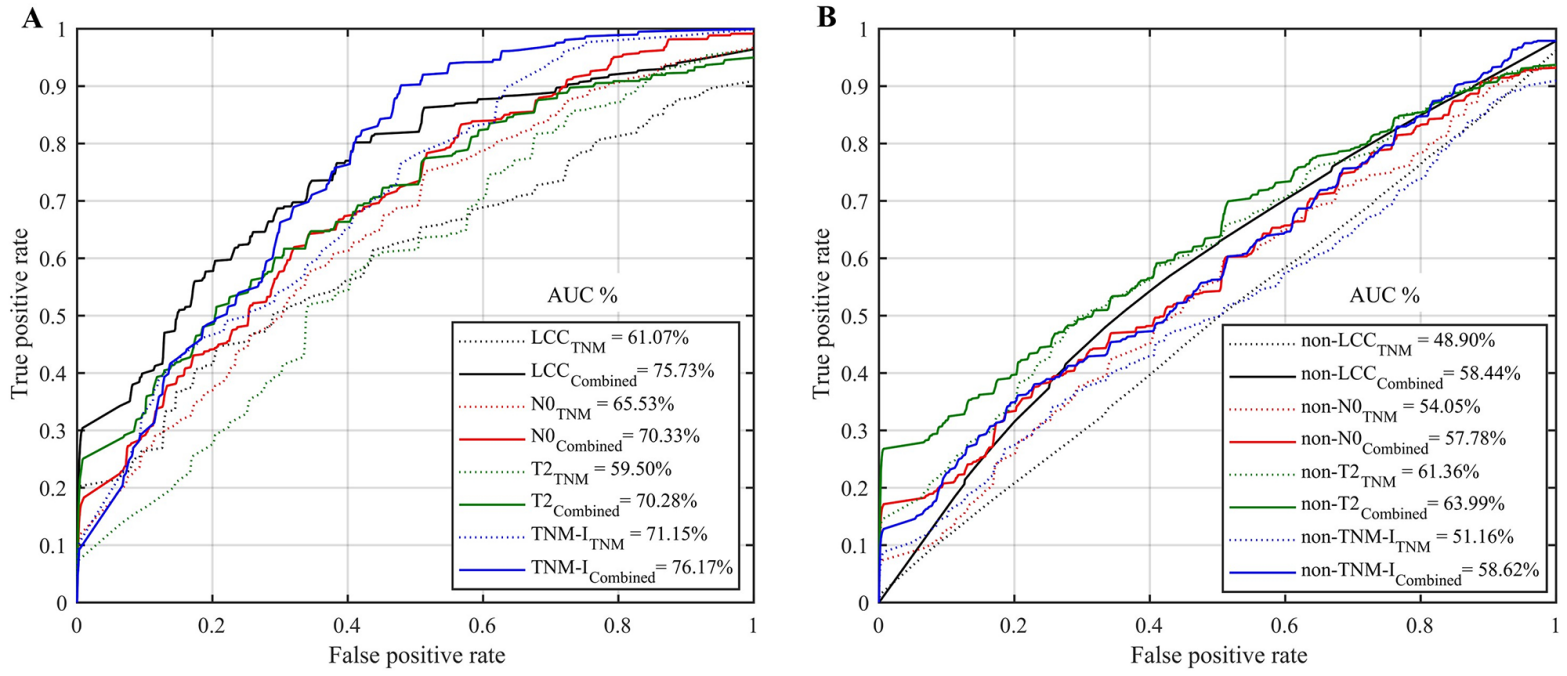
Mean ROC curves and AUC obtained by the random forest models for predicting the survival group (i.e., below or above median survival time) using only demographic and TNM staging information (TNM), or combined with radiomic features (Combined) **(A)** Patients group of large cell carcinoma (LCC, n=100), N0 (n=133), T2 (n=120) and TNM-I (Stage I, n=81). **(B)** Patients group of non-LCC (i.e., n=177: squamous cell carcinoma, adenocarcinoma and not otherwise), non-N0 (i.e., n= 182: N1, N2, N3 and N4), non-T2 (i.e., n=192: T1, T3 and T4) and non-TNM-I (i.e., n=233: Stage II, IIIa and IIIb).

Figure 5A shows the mean ROC curves and AUC values obtained by the RF models for predicting the survival outcome (i.e., below or above the median survival time) of patients within the LCC, T2, N0, Stage I groups. Note that these groups were previously shown to exhibit moderate correlations with survival. To better assess the individual effect of these groups, Figure 5B gives the prediction AUC obtained for subjects not in these groups, i.e. non-LCC (SCC, ADC and NOS), non-T2 (T1, T3 and T4), non-N0 (N1, N2, N3, and N4) and non-TNM-I (TNM groups: II, IIIa and IIIb) subjects. We compare predictive models based only on demographics and TNM staging information (5 features: age, T, N, M, and Stage) or combined with radiomic features (24 features: texture and shape), as indicated by the eight ROC curves.

We see that combining radiomic features with age-TNM information generally leads to improved prediction, with an average AUC of 75.73%, 70.33%, 70.28%, 76.17% compared to 61.07%, 65.53%, 59.50%, 71.15% when using only age-TNM information, for LCC, N0, T2, and Stage I patient groups respectively (Figure 5A). Moreover, we find that combining radiomic features with age-TNM information generally leads to improved predictions, with an average AUC of 58.44%, 57.78%,63.99%, 58.62% compared to 48.90%, 54.05%, 61.36%, 51.16% when using only age-TNM information, for non-LCC, non-N0, non-T2, and non-TMI-I patient groups respectively (Figure 5B). Considering all groups (i.e., LCC, T2, N0, Stage I, non-LCC, non-T2, non-N0 and non-TNM-I), radiomic features combined with age-TNM information lead to the highest AUC value of 76.17% for Stage I patients.

**Figure 6:**
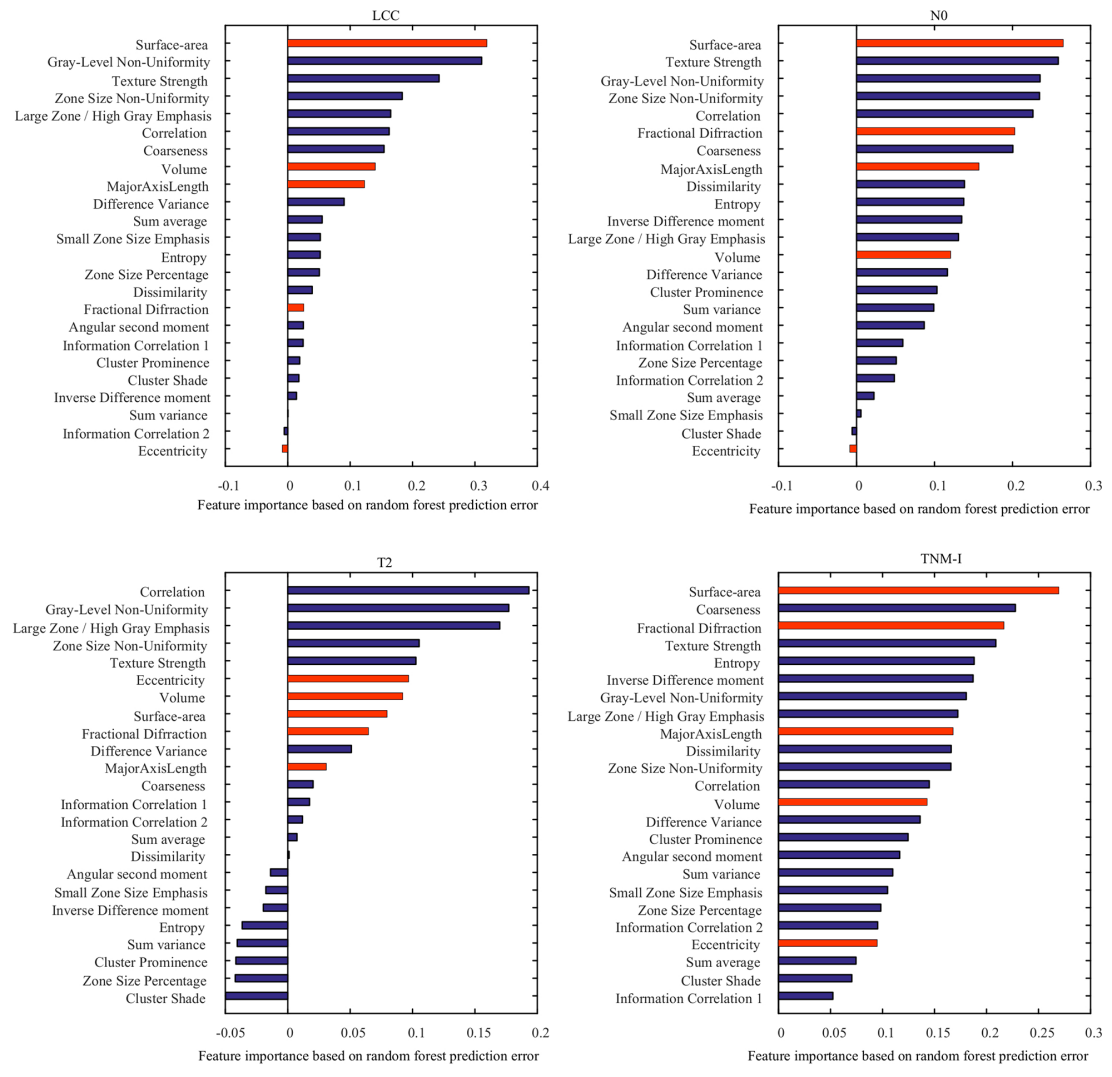
Importance of individual features for predicting the survival group LCC, T2, N0 and TNM-I (stage I) patients Reported values correspond to the mean increase in prediction error obtained by permuting the values of individual features across out-of-bag observations [49]. Blue and red bars represent texture and shape features, respectively.

Results of our feature importance analysis are presented in Figure 6. Features identified as important are consistent with those identified using Spearman’s rank correlation and log-rank test. In particular, surface-area and grey-level non-uniformity were ranked as the most common discriminative features across LCC, T2, N0, and Stage I patient groups. In addition, we observe that both texture and shape features are informative to differentiate patients with short and long survival time.

## DISCUSSION

Current tools for predicting survival of NSCLC patients are based primarily on clinical and staging information. Nomograms for predicting patient survival from gene expression signatures, clinical and pathological features are not yet ready to be used in daily practice. Radiomic features extracted from CT scans provide a non-invasive and powerful alternative for identifying prognostic or predictive biomarkers of survival in cancer patients. This study performed three different analyses to evaluate the usefulness of radiomic features for predicting the survival outcome of patients with specific NSCLC cancer subtypes and stages.

Our analysis based on Spearman’s rank correlation identified several radiomic features that were moderately correlated with the survival outcome of patients with LCC cancers, T2 tumor sizes, or classified as Stage I. These correlation results were statistically significant with *P* < 0.05 following Holm-Bonferroni (Figure 1/Figure 2). Furthermore, log-rank testing revealed four texture features exhibiting significant associations with survival for the same patient groups (i.e., LCC, T2 and Stage I patients). Finally, our multivariate analysis using random forest models showed the potential of radiomic biomarkers for predicting the survival outcome of NSCLC patients (Figure 5), in particular, those in the LCC, T2, N0 and Stage I groups.

These findings are consistent with previous works in the literature, which have found various texture and shape features to be strong predictors of NSCLC survival outcome [17-19, 30-34]. In contrast to these works, this study analyzed the link between radiomic features and survival for specific NSCLC cancer subtypes and stages. Our results suggest that radiomic features might be more relevant from survival prediction in the case of large cell carcinoma cancers with a primary tumor between 3 cm and 7 cm across, no lymph node involvement, and without metastasis. Since features are extracted from the primary tumor only, this could potentially be explained by the fact that small tumors (i.e., less than 3cm across) provide limited texture and shape information, compared to larger ones, and that the impact of this tumor on outcome is less important once lymph nodes are affected or the cancer has metastasized to other organs.

While previous studies have found tumor shape to be a good predictor of NSCLC patient survival [35, 36], our experiments indicate that texture features may be more effective at this task. In particular, NGTDM features corresponding to texture coarseness and strength, as well as GLZM features based on zone size non-uniformity and grey-level non-uniformity, appear to be suitable predictors of overall survival. Since these features are relatively easy to compute, they could be used in a clinical setting to establish prognosis. It is worth mentioning that texture features are usually more sensitive to image acquisition equipment and parameters than those based on shape. Because our study uses CT images, and the intensities in such images are determined by the radiodensity of scanned tissues (i.e., Hounsfield units), the influence of acquisition variables on radiomic features is limited. Nevertheless, normalizing image intensities or learning predictive models specific to a particular equipment could therefore help provide consistent results across patients.

As in previous work [7, 9], our analysis has shown radiomic features to improve the prediction of NSCLC survival compared to using only TNM staging information. It is hypothesized that texture features can capture tissue anomalies occurring at the cellular level that are directly related to cancer subtype and stage. Likewise, shape features could describe the irregularity of NSCLC tumors during their progression, which may vary for different cancer subtypes or stages. This motivates our approach of analyzing cancer subtypes and stages separately, unlike previous studies.

Our study has some limitations worthy of mention. In our correlation and log-rank survival analyses, differences in group sizes may affect significance values. It is thus possible that some results of non-significance are due, in part, to small group sizes. This could be addressed in a future work by using a larger patient cohort. Patients in different groups could also be matched, for instance, to remove age and gender bias. Although we used a 10-fold cross validation strategy to obtain unbiased estimates of prediction accuracy, experiments using additional independent datasets would further validate our proposed method, in particular NSCLC data acquired from multiple sites and imaging modalities other than CT. In addition, patient survival is generally related to a variety of factors, including treatment, psychology, diet, etc. that were unavailable in the TCIA data set for analysis. These factors may potentially introduce bias into our results, however this bias is reduced by size of our patient cohort (315 patients). Using labels from multiple raters, instead of a single one (i.e., the radiation oncologist), could also reduce bias in the results.

In our survival analysis, the median survival time was used as cut-off to divide patients in two subgroups (i.e., classes) corresponding to short and long survival. This strategy has the important advantage of giving even-sized subgroups, thereby eliminating the bias introduced by class-unbalanced samples. However, it is also limited in that biologically similar patients about the cutoff threshold (i.e. median age) are grouped into different categories, which may negatively impact analysis [37]. In the proposed analysis, this problem is mitigated in part via a 10-fold cross-validation methodology, where prediction accuracy is measured over ten independent data subsamples.

Since our results indicate that radiomic features from the primary tumor have lower predictive power when lymph nodes are affected, a logical extension of this work would be to add features extracted from metastatic lymph nodes in lung CT scans. Finally, machine learning techniques such as convolutional neural networks [38] could be employed to learn discriminative features in a more data-driven manner.

In conclusion, this study demonstrated the potential of radiomic features capturing textural and morphological properties of NSCLC tumors as non-invasive biomarkers to predict the survival outcome of NSCLC patients. In contrast to previous works, we analyzed the association between radiomic features and survival for patients within specific groups defined by cancer subtype and stage. Results suggest that these features are mainly relevant in the case of large cell carcinoma cancers (LCC) with primary tumor size between 3 cm and 7 cm across (T2) and no lymph node metastasis (N0). Results also highlight the need for future studies including radiomic features extracted from lymph nodes metastasis.

## MATERIALS AND METHODS

**Figure 7:**
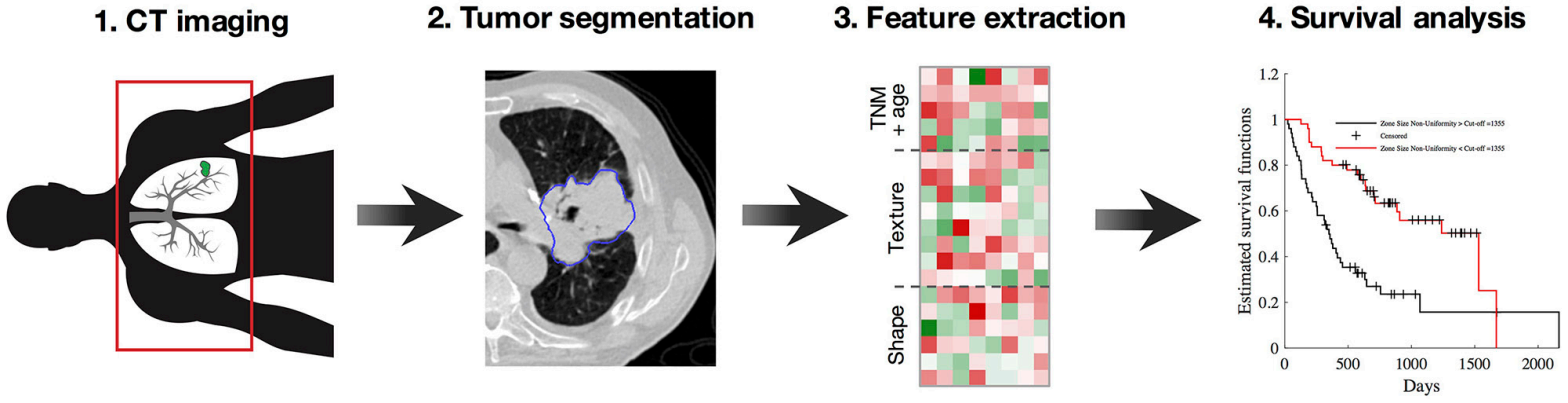
Workflow of the proposed radiomics method (i.e., our pipeline, similar to [6, 7]) **(1)** Acquisition of pre-treatment lung CT images; **(2)** Gross tumor volume segmentation and NSCLC subtype labelling; **(3)** Extraction of 24 texture and shape features from gross tumor volumes; **(4)** Feature significance analysis based on Spearman rank correlation, Kaplan-Meier estimator and log-rank test, and multivariate prediction of survival using random forests.

Figure 7 shows the processing pipeline of the proposed method. Raw imaging data from patients with NSCLC cancer are first acquired by CT scan, prior to treatment. For each scan, the gross tumor volume (GTV) is then computed from manual delineations provided by a radiation oncologist, and assigned to one of four the NSCLC subtypes (i.e. large cell carcinoma (LCC), squamous cell carcinoma (SCC), adeno-carcinoma (ADC) or not otherwise specified (NOS)). The oncologist also classifies tumor progression based on the tumor-node-metastasis (TNM) staging system [39], describing the size and invasion level of the tumor, the presence of affected lymph nodes, and whether the cancer has metastasized to distant organs.

A total of 24 features (i.e. 19 texture features and 5 shape features) are then automatically computed from extracted GTVs, and used in combination with patient age to analyze the survival outcome of patients. Three separate analyses were conducted toward this goal. In the first analysis, Spearman rank correlation was used to measure the relationship between each feature and survival time. Similarly, the second analysis uses the Kaplan-Meier estimator and log-rank test to find features leading to significantly different survival curves when dividing subjects based on the features’ median value. For these two analyses, feature significance is reported in terms of p-values, corrected for multiple-comparisons using the Holm-Bonferroni procedure [40]. Finally, a multivariate analysis based on the random forest model is employed to classify NSCLC patients into groups corresponding to short survival (i.e. below the median survival time) and long survival (i.e., above or equal to the median survival time). For all analyses, we used various toolboxes from the MATLAB 2016 computing environment (MathWorks Inc., Natick, MA, USA).

### Patients and data acquisition

Analysis involves the subset of 315 patients with complete NSCLC labels, from the set of 422 patients in the *The Cancer Imaging Archive* (TCIA, http://cancerimagingarchive.net/) [41]. This dataset, called Lung1, contains data of patients treated at the MAASTRO Clinic, Netherlands, previously de-identified by the Cancer Genome Atlas (TCGA, http://cancergenome.nih.gov/) and made publicly available for download. Thus, no institutional review board approval specific to this study was required. All images were acquired using CT scan at a resolution of 512×512×slices, where the number of slices varied across subjects, and a voxel size of 1×1×1 mm^3^. For each scan, the gross tumor volume (GTV) was manually delineated by a radiation oncologist and provided as segmentation mask. A subset of 277 cases were also classified by the oncologist based on the standard TNM staging system, measuring the tumor size (T), the extent of regional lymph node involvement (N) and the presence or absence of intrathoracic or distant metastases (M). Finally, the survival time in days, from time of scan to death (i.e., uncensored) or last visit (i.e., censored), was also provided for all 315 patients. Patient demographic information (i.e., gender and age) for each NSCLC subtype and TNM parameter is reported in Table 1.

### Feature extraction

A wide variety of radiomic features may be computed from the region of interest (i.e., the GTV in our case). In this study, we focused on a subset of 24 commonly used texture and shape features, which are presented in Supplementary Table 1 of the Supplementary Materials. Three different types of texture features were considered: grey level co-occurrence matrix (GLCM), neighborhood grey-tone difference matrix (NGTDM), and grey-level zone matrix (GLZM). These features measure various textural properties of the GTV, such as region uniformity/heterogeneity and texture coarseness, which were shown to be related to histological properties of tumors [42, 43]. To capture more meaningful patterns of texture, image intensities of GTVs were uniformly resampled to 32 grey-levels prior to computing the features. On the other hand, shape features encode morphological characteristics of the tumor, such as volume and surface area, that capture the tumor growth status within surrounding tissues [44].

### Statistical analysis

In the first analysis, we computed the Spearman’s rank correlation [45] between the features extracted from each GTV and the survival time of the corresponding patient. For censored patients, the time of last visit only offers a lower bound on the true survival rank. To account for these patients in our correlation analysis, we used a simple imputation strategy in which censored patients are assigned the mean survival time of uncensored subjects with a time-to-death greater or equal to their own time of last visit. Using this strategy, rank correlation was obtained between the survival time of patients and each radiomic feature (plus patient age), absolute values between 0.3 and 0.5 indicating moderate correlation. Additionally, the significance of these correlation values was measured as p-values, based on the null hypothesis that there is no correlation.

The relation between radiomic features derived from the GTV and patient survival possibly depends on the NSCLC subtype or the cancer’s stage. For instance, such features may be less informative in patients with affected lymph nodes or metastasis, these factors becoming more important than the primary tumor for overall prognosis. To validate this hypothesis, we repeated our analysis on various patient groups, corresponding to different NSCLC subtypes and TNM variable classes. To account for these multiple comparisons (e.g., 4 NSCLC subtypes and 24 features + age, for a total of 100 tests), we corrected the p-values of our analysis using the Holm-Bonferroni procedure [40], and considered as significant results with corrected *P* < 0.05.

In the second analysis, we considered each feature in turn and used the median value of this feature to separate patients in two groups: those with feature value less than the median, and those with feature value above or equal to the median. As in [46], we then computed the time-to-event (i.e., number of days from scan until death or last visit) distributions of the two groups using the Kaplan-Meier estimator, and compared them using the log-rank significance test. The same patient groups as in the previous analysis were considered, and p-values were corrected based on the same procedure.

Furthermore, we performed a multivariate analysis using all 24 radiomic features and 5 demographic/staging variables (i.e., age, T, N, M and overall stage) as input to a Random Forest (RF) model [47] for the classification of patients in two groups representing short survival (i.e., below the median survival time) and long survival time (i.e., above or equal to the median survival time). RF is one of the most effective and general-purpose classification algorithms, running efficiently on large databases with thousands of input variable/features. This model operates by averaging the output of a battery of randomly generated decision tree classifiers, a general technique known as bootstrap aggregation which leads to a low bias/variance classification result. Additionally, the RF training algorithm involves a feature selection process that provides a mechanism for assessing feature importance.

The hypothesis for this analysis is that radiomic features can improve survival prediction, compared to demographics and TNM staging information. As in the correlation analysis, censored patients were considered via an imputation strategy, where the mean survival of uncensored subjects with time-to-death greater or equal to the time of last visit was used. Likewise, we predicted survival considering the same patient groups as in the correlation analysis to determine the impact of these grouping parameters on performance. A 10-fold cross-validation strategy was employed to obtain unbiased performance measures. In this strategy, data samples of every patient group were randomly divided into 10 even-sized sets (i.e., folds). Each of these sets was then used, in turn, to compute the area under the ROC curve (AUC) [48] of a RF model trained with the remaining samples, using 500 decision trees. The overall performance of the model was then measured as the average AUC obtained over all 10 folds.

Finally, the importance of each feature in predicting the survival group of patients was assessed based on the out-of-bag error of the multivariate RF models generated at each fold. Specifically, for each RF model and feature, we measured the increase in prediction error resulting from the permutation of feature values across out-of-bag observations [49]. These importance measures were computed for every RF tree and averaged over the entire ensemble. Values were then normalized by dividing them by the ensemble’s standard deviation. Lastly, the importance of features was obtained by averaging these normalized values across all 10 folds.

## SUPPLEMENTARY MATERIALS AND TABLE




