# Supplementary material for: Predicting survival time of lung cancer patients using radiomic analysis
Source: Oncotarget. 2017 Nov 1;8(61):104393–407. doi: 10.18632/oncotarget.22251 (PMC5732814; doi:10.18632/oncotarget.22251)
Supplement: Supplementary file 1 [file oncotarget-08-104393-s001.pdf]

Predicting survival time of lung cancer patients using radiomic analysis

SUPPLEMENTARY MATERIALS

Grey-level co-occurrence matrix (GLCM) [1]**:** These texture features use second order statistics to characterize the spatial relationship between intensity values within a 3D volume. Specifically, a 3D GLCM P_d gives the joint probability $p_d(i, j)$ of intensities values i and j in a pair of voxels separated by a displacement vector $d = [d_x\ d_y\ d_z]$ [1, 2]. In this work, we uniformly resampled image intensities to 32 grey-levels and generated 13 GLCMs based on displacements corresponding to the 3^3-1 = 26 neighborhood of voxels. Note that, since $p_d(i, j)$ is symmetric, only 26/2 = 13 displacements need to be considered. We then encoded each of these GLCMs using 12 quantifier functions proposed by Haralick [1]: angular second moment, correlation, inverse difference moment, sum average, sum variance, entropy, difference variance, information correlation 1, information correlation 2, dissimilarity, cluster shade and cluster prominence. These quantifier functions are described in Supplementary Table 1. Finally, the texture features of each GTV were obtained by averaging the 12 quantifier function values over the 13 GLCMs.

Neighborhood grey-tone difference matrix (NGTDM) [3]: These features also use higher-order statistics to measure intensity differences between neighbor voxels. Let $\bar{a}(x, y, z)$ be the average intensity value in the neighborhood of a voxel coordinate (x, y, z) excluding this voxel. NGTDMs are defined using a vector s such that

$$s(i) = \sum_{(x,y,z)\in N_i} \left| i - \bar{a}(x,y,z) \right|, \quad (1)$$

where N_i is the set of voxels with intensity i. Furthermore, NGTDM features also use a vector p where $p\ (i)$ is the probability of intensity i in the volume (i.e. the number of voxels with intensity i over the total number of voxels). As described in Supplementary Table 1, we use s and p to compute two features measuring the coarseness and strength of texture within the GTV.

Grey-level zone size matrix (GLZM) [4]**:** These texture features characterize the size of uniform voxel regions, called zones. GLZM features are based on a run-length matrix M defined such that $m\ (s, i)$ gives the number of connected zones in the volume, containing s voxels with intensity i. Using these, we extracted five features describing the homogeneity of GTVs: small zone size emphasis, large zone/high grey emphasis, grey-level non-uniformity, zone size non-uniformity and zone size percentage. Details about these features can be found in Supplementary Table 1.

Shape features [5, 6]: As presented in Supplementary Table 1, we also extracted five shape features encoding morphological properties of GTVs: major axis length, eccentricity, volume, fractal dimension, and surface-area. While the first three encode information about the tumor's size and shape, the last two characterize the smoothness of its surface. Additionally, we include age as a clinical feature (f_{25}).

REFERENCES

1. Haralick RM. Statistical and structural approaches to texture. Proc IEEE. 1979; 67: 786–804.
2. Gao X, Qian Y, Hui R, Loomes M, Comley R, Barn B, Chapman A, Rix J. Texture-based 3D image retrieval for medical applications. IADIS International Conference e-Health [Internet]. 2010 [cited 2016 Oct 16]. p. 101–108. Available from http://image.mdx.ac.uk/mirage2011/ehealth_2010.pdf.
3. Amadasun M, King R. Textural features corresponding to textural properties. IEEE Trans Syst Man Cybern. 1989; 19: 1264–74. https://doi.org/10.1109/21.44046.
4. Thibault G, Fertil B, Navarro C, Pereira S, Cau P, Levy N, Sequeira J, Mari JJ. Texture indexes and gray level size zone matrix application to cell nuclei classification. 2009 [cited 2016 Oct 16];. Available from http://elib.bsu.by/handle/123456789/51362.
5. Yang X, Beyenal H, Harkin G, Lewandowski Z. Quantifying biofilm structure using image analysis. J Microbiol Methods. 2000; 39: 109–19. https://doi.org/10.1016/S0167-7012(99)00097-4.
6. Legland D, Kiêu K, Devaux M-F. Computation of Minkowski measures on 2D and 3D binary images. Image Anal Ster. 2007; 26: 83–92.

Supplementary Table 1: Description of features used in the analysis

See Supplementary File 1
