# Supplementary material for: Predicting survival time of lung cancer patients using radiomic analysis
Source: Oncotarget. 2017 Nov 1;8(61):104393–407. doi: 10.18632/oncotarget.22251 (PMC5732814; doi:10.18632/oncotarget.22251)
Supplement: Supplementary file 2 [file oncotarget-08-104393-s002.docx]

Supplementary Table 1: Description of features used in the analysis

| **Features** | | **Formula / Description** |
| --- | --- | --- |
| **Grey-level co-occurrence matrix (GLCM)** | | |
| *f_1_* | Angular second moment (Energy) | $\sum_{i=1}^{N} \sum_{j=1}^{N} {p(i,j)}^{2}$ |
| *f_2_* | Correlation | $\frac{1}{\sigma_{x}\sigma_{y}}\sum_{i=1}^{N} \sum_{j=1}^{N} {\left( ij \right).p(i,j)}^{2}-\mu_{x}\mu_{y}$ |
| *f_3_* | Inverse difference moment | $\sum_{i=1}^{N} \sum_{j=1}^{N} \frac{1}{1+\left( i-j \right)^{2}}.p\left( i,j \right)$ |
| *f_4_* | Sum average | $\sum_{i=2}^{2N} i.p_{x+y}(i)$ |
| *f_5_* | Sum variance | $\sum_{i=2}^{2N} \left( i-f_{6} \right)^{2}{.p}_{x+y}(i)$ |
| *f_6_* | Entropy | $-\sum_{i=1}^{N} \sum_{j=1}^{N} p\left( i,j \right).log(p\left( i,j \right))$ |
| *f_7_* | Difference variance | $\sum_{i=0}^{N-1} {i^{2}.p}_{x+y}(i)$ |
| *f_8_* | Information correlation 1 | $\frac{HXY-{HXY}_{1}}{max(HX,HY)}$ |
| *f_9_* | Information correlation 2 | $(1-{\exp\left( -2\left\vert{HXY}_{2}-HXY \right\vert\right))}^{2}$ |
| *f_10_* | Dissimilarity | $\sum_{i=1}^{N} \sum_{j=1}^{N} \left\vert i-j \right\vert.p(i,j)$ |
| *f_11_* | Cluster shade | $\sum_{i=1}^{N} \sum_{j=1}^{N} {(i+j-\mu_{x}-\mu_{y})}^{3}.p(i,j)$ |
| *f_12_* | Cluster prominence | $\sum_{i=1}^{N} \sum_{j=1}^{N} {(i+j-\mu_{x}-\mu_{y})}^{4}.p(i,j)$ |
| **Note:** *P* is the 3D GLCM matrix; *μ_x_, μ_y_,σ_x_,* *and σ_y_,* are the mean and standard deviations of partial probability density functions *p_x_* and *p_y_;HXY* is the entropy of *P* (i.e., *f_6_*); HX and HY are the entropy of *p_x_* and *p_y_*;  HXY_1_= $-\sum_{i=1}^{N} \sum_{j=1}^{N} p\left( i,j \right).log(p_{x}\left( i \right)p_{y}\left( j \right))$; HXY_2_= $-\sum_{i=1}^{N} \sum_{j=1}^{N} p_{x}\left( i \right)p_{y}\left( j \right).log(p_{x}\left( i \right)p_{y}\left( j \right))$ | | |
| **Neighborhood grey-tone difference matrix (NGTDM)** | | |
| *f_13_* | Coarseness | $\left[ \sum_{i=1}^{N} p\left( i \right).s(i) \right]^{-1}$ |
| *f_14_* | Texture Strength | $\frac{\sum_{i=1}^{N} \sum_{j=1}^{N} (p\left( i \right)+p\left( j \right)).{(i-j)}^{2}}{\sum_{j=1}^{N} s(i)}$ |
| **Note:** *s* is the NGTDM vector; *p* is the grey-level probability vector. | | |
| **Grey-level zone size matrix (GLZM)** | | |
| *f_15_* | Small zone size emphasis | $\frac{1}{N_{r}}\sum_{i=1}^{N} \sum_{s=1}^{S} \frac{m(s,i)}{s^{2}}$ |
| *f_16_* | Large zone/high  gray emphasis | $\frac{1}{N_{r}}\sum_{i=1}^{N} \sum_{s=1}^{S} \frac{m\left( s,i \right).s^{2}}{i^{2}}$ |
| *f_17_* | Gray-level  non-uniformity | $\frac{1}{N_{r}}\sum_{i=1}^{N} {(\sum_{s=1}^{S} m\left( s,i) \right)}^{2}$ |
| *f_18_* | Zone size  non-uniformity | $\frac{1}{N_{r}}\sum_{s=1}^{S} {(\sum_{s=1}^{S} m\left( s,i) \right)}^{2}$ |
| *f_19_* | Zone size  Percentage | $\frac{N_{r}}{m\left( s,i \right).s}$ |
| **Note:** *m* is the GLZM matrix; *N_r_* is the total number of unique connected zones. | | |
| **Shape features** | | |
| *f_20_* | Major axis length | Length (in voxels) of the major axis of an ellipse with same normalized second central moments as the GTV. |
| *f_21_* | Eccentricity | Ratio of the distance between the foci and the major axis length of an ellipse with same second-moments as the GTV. |
| *f_22_* | Volume | Number of voxels in the GTV. |
| *f_23_* | Fractal dimension | $\lim_{\varepsilon\to0} \frac{ln(N(\varepsilon))}{ln(1/\varepsilon)}$ , where *N*($\varepsilon$) is the number of cube of side $\varepsilon$ necessary to cover the GTV. |
| *f_24_* | Surface-area | Area of the GTV’s surface based on the Cauchy-Crofton formula [1] |

1. Li X, Wang W, Martin RR, Bowyer A. Using low-discrepancy sequences and the Crofton formula to compute surface areas of geometric models. Comput- Aided Des. 2003; 35: 771–82. https://doi.org/10.1016/ S0010-4485(02)00100-8.
